# The Covid-Shock Doctrine: Under the Tutorship of CoV-2, the Voice(s) From Poland

**DOI:** 10.1177/1532708620931144

**Published:** 2021-04

**Authors:** Oskar Szwabowski, Marek Wiecław

**Affiliations:** 1 Uniwersytet Szczecinski, Poland

**Keywords:** shock doctrine, neoliberalism, autoethnography, ethnographies, methodologies, coronavirus

## Abstract

These are two autoethnographic voices. We speak in a strange time: democracy dies, social justice dies. A lot of people have died of the virus, many die of fear. We write to protest against new neoliberal and neoconservative “shock doctrine.” We write together to protest against destructive self-absorption, isolation, and fear. It is protest-text. But we are not sure what we can do now.

## Intro

It is a strange time. I do not know what will be tomorrow. I have to stay at home. I do not know how much I will earn and what is happening around. I feel that there is the opportunity to change our social reality for a better place. But it can be changed also to the rebirth of the neoliberalism regime—make it crueler.

What we can do? How we can survive?

I try to speak. I have a lot of time, but it is not a good time for research. For thinking also. So I write such not a scientific paper. It is a note from epidemics. I write because I feel alone. I feel helpless. I feel like a felt before. And I write for the same reasons. As Ronald J. Pelias wrote—to be here, still, to be part of conversation (Pelias, 2017). And because “I write so the darkness cannot win” (Poulos, 2017, p. 38).

So I am writing to be alive—somehow—and try to make a difference.

In my country, now the situation is not so bad. We are still waiting. But the virus of neoliberalism spreads faster and intensely. It can bring more damage to our society than Coronavirus. How we can react to that when we have to stay at home? How we can protect and promote social justice and the poor? Could we use this Coronavirus as a partner in making the world a better place?” What are we taught now?

And how can we win with isolation . . . with our dark lonely thoughts.

To struggle with our destructive self-absorption, fear, and isolation, what makes us vulnerable? We are immersed in our own sad self. We are overcome by discouragement. We don’t have the strength to protest. For what if the world will end soon.

The doctrine of shock works (Klein, 2008).

I send this text to my friend. He joins me.

Marek:

Writing in Time of Plaque?

What a concept.

Oskar:

Not sure what we can do more.

Marek:

How can I think to have Anything Original to tell you in time-like-this?

And why do I need to be original anyway?

Oskar:

We do not have to be original. I know that being original is of some value for “normal science”. But now “world is out of joint”. We write to protest. Not to be original. Not to be a super academic researcher. I think this time is the end for humanities and social science.

## I

Stay, stay away . . .

  borders are closed

       troops with guns protect you.

   They defend our country against immigrant, strangers are the virus

       They will kill you. We and God will protect you.

Trust us.

Stay away. . .

people are dangerous

government will protect you

trust us

         or else . . .

my grandma said to me—this virus is dangerous only for foreigners

  do not worry

     stay away

        stay at home

be responsible

everything is up to you

“We lack everything:

        masks,

             glasses,

                  helmets,

     disinfectants”^1^

“resources are shrinking at an alarming speed”

(TVN24, 2020).

uncertainty and fear

old story in a new setting

another scene

show how

neoliberalism leads us

to extinction

## II

Dear Coronavirus

I write to you with hope

You can teach us a lot

        to not eat animals

        and

        how we are connected

        how important is to have good public healthcare

        how cruel are temporary contracts

        how many activities our pointless

           we do not travel so much

             we do not go shopping so often

              we can joy simple things like being together

                and being alive

but

no

no no no no

nothing changes

just

intensification of necrophilics neoliberalisation (Gounari, 2014, 2016; Mendoza, 2015)

social Darwinism

disdain for the weakness

junk contracts—junk works—junks workers

just—all time

all well know anti-public pedagogy (Giroux, 2011; Morris, 2012)

   junk-peoples

## III

Few days ago, I read “Letter from your Future” by Italian novelist. She lives in our future—in the world that is probably one month ahead of us.

So she sends us the message. This voice of her—the voice from the City of Plaque, from the Future—has been following me since then.

“As we watch you from here, from your future, we know that many of you,

as you were told to lock yourselves up into your homes,

quoted Orwell, some even Hobbes.

But soon you’ll be too busy for that” (Melandri, 2020)

My friend calls me

      every day

today more people die

today more people are sick

I do not feel well

My friend calls me

 every day

    day by day

it is getting worse

everybody in my flat feel anxiety

    how long can it take?

it had to explode

today more people die

today more people are sick

“resources are shrinking at an alarming speed”

(TVN24, 2020).

Your own thoughts can be annoying. Haven’t you noticed this before?

Obsessed And I became tired even of my own favorite dark sarcasm.

*“You’ll find dozens of social networking groups with tutorials*

*on how to spend your free time in fruitful ways.*

*You will join them all, then ignore them completely*

*after a few days.”*(Melandri, 2020)

What matters now?

## IV

I know I am lucky

I am white, I am man, I am low middle classes academic workers

I can work from home

and still have my job and my home

I am quite young and quite healthy

so I should not be worried

and

it taught me that

disease is a social class problems

it is political and economic problems

“The black week is coming.

Even 3 million Poles may lose their jobs”

(Ceglarz, 2020)

“employees and employees will pay

for the crisis

(again)”

(OZZIP, 2020)

“The government wants to change Poland into a labor camp” (Urbański, 2020)

“Democracy is dead, long live the pandemiocracy”

(Bendyk, 2020)^2^

On facebook words flows. . .

“We have a coup d’état.

   under the guise of coronavirus

      and the worst is powerlessness

I sit and cry

from

anger

pain

despair

our anger

Please

wake up!”

## VI

Back from the kitchen. During the Plaque Quarantine you eat a lot.

You watch silly movies.

You try to murder Goethe and Wittgenstein.

You don’t even look at *Finnegan’s Wake* (ok, you don’t even know where it is now.

And you don’t care).

*“You’ll pull apocalyptic literature out of your bookshelves*, *but will soon find you don’t really feel like reading any of it”* (Melandri, 2020)

I got survey form sociologists—it is a unique situation, tell us how you live –

        wow!

        new data new thing

        to write about it

        and become famous

        or just curious

        how you suffer

            Of course it is on-line survey

            just for people who have home

            computer and internet

I am thinking about what I can do now as a critical educator and critical qualitative researcher

           How can we help coronavirus to change world for a better place and kill neoliberalism for good?

Yesterday

my anarchist friends made masks for people

            no one spoke about revolution

Now

  We cannot leave home if we do not have good reasons

            Read: go to work, go to shop

               work-consume-die

I cannot speak with homeless

      with alone old people

   the pedagogy of street is not possible anymore (Lewis, 2012; Szwabowski, 2019)

## VII

I have tried to search for some rational scientific explanation the other day.

What the hell is happening to the world anyway?

Who did this? Why?

Anyone?

I spent two days at the computer surfing network.

Articles, films, diagrams, tables, statements, declarations.

Doubts, questions.

NOTHING. NOBODY.

It is just us.

*“You will not sleep well.*

*You will ask yourselves what is happening to democracy.”* (Melandri, 2020)

as humanists and social scientists we can cure our social disease

perhaps we will beat Coronavirus

but social death remain with us

unless we beat

capitalism and this permanent state of exception (Agamben, 1998)

and fascists governments

the anti-public pedagogy (Giroux, 2011; Morris, 2012) and the pedagogy of disdain (see. Szkudlarek, 2018).

in this shadow of death and pathological neoliberalism

social Darwinism fascist (Giroux, 2011, 2018)

we have to dream radically

normality will not be back

it is our new normality

we have to

find the way to be together

to make our dreams publics

is it time for the rebirth of public intellectual in old style?

or

maybe it is time for collective democratic writing

to build new world on collective experience and dreams

of course

with people who have home

computers

internet

and time

## Notes

1. Kamil Barczyk, director of the hospital in Bolesławiec (in Rybak, 2020, March 10).

2. At the night of March 28, 2020, rules party changes the rules for participation in upcoming elections.
